# Draft Genome Sequence of Spiroplasma platyhelix ATCC 51748, Isolated from a Dragonfly

**DOI:** 10.1128/MRA.00422-20

**Published:** 2020-11-19

**Authors:** Emily A. Green, Jonathan L. Klassen

**Affiliations:** a Department of Molecular and Cell Biology, University of Connecticut, Storrs, Connecticut, USA; Indiana University, Bloomington

## Abstract

Spiroplasma platyhelix is a helical bacterium belonging to the class *Mollicutes*. First isolated from a *Pachydiplax longipennis* dragonfly, it has the smallest reported *Spiroplasma* genome size of 740 kbp. Here, we report the genome sequence of *S. platyhelix* ATCC 51748.

## ANNOUNCEMENT

Spiroplasma platyhelix is a species of *Mollicutes* bacteria that was first isolated from a Pachydiplax longipennis dragonfly (1). It has the smallest genome size of all known *Spiroplasma* species, bacteria that infect insects, animals, and plants, acting as pathogens or mutualists (1). Bacteria related to *S. platyhelix* have been found in several fungus-growing ants (2, 3) and field crickets (NCBI accession number JQ768460).

*S. platyhelix* ATCC 51748 (synonym: strain PALS-1) was purchased from the ATCC and grown in SP-4 medium (4) at 30°C for 7 days in a 15-ml screw-top Falcon tube. Genomic DNA was extracted using the Epicentre MasterPure Complete DNA and RNA purification kit following the DNA purification protocol for cell samples. The DNA concentration was determined using a Qubit high-sensitivity assay. Libraries were prepared from the same DNA extract for whole-genome shotgun sequencing using both 2 × 150-bp and 2 × 250-bp Nextera library kits, following the manufacturer’s protocol, and sequenced on an Illumina MiSeq instrument. Adapters were removed using Trimmomatic v0.39 (5), and data were quality checked using FastQC v0.11.8 (https://www.bioinformatics.babraham.ac.uk/projects/fastqc/). A Nanopore MinION long-read sequencing library was prepared from this extract following the native barcoding genomic DNA (with EXP-NBD104, EXP-NBD114, and SQK-LSK109) protocol (vNBE_9065_v109_revV_14Aug2019). Modifications included using a NEBNext companion module for the Oxford Nanopore Technologies ligation sequencing kit for ligation, omitting shearing and size selection and not using long fragment buffer during adapter ligation and cleanup. The library was sequenced on an MK1B MinION device using R9.4 flow cells and using MinKNOW v19.12.2 to run MinKNOW core v3.6.0 and Guppy v3.2.8 for base calling, demultiplexing, and barcode trimming. Reads were assembled using SPAdes v3.13.0 (6) and annotated using Prokka v1.13.3 (7). Genome completeness was checked against the BUSCO *Tenericutes* database (8), and average nucleotide identities (ANIs) were calculated using the ANI Calculator (https://www.ezbiocloud.net/tools/ani) (9). All analyses used default parameters.

In total, 332,773 and 326,607 reads were obtained for the 2 × 150-bp and 2 × 250-bp libraries, respectively, and 2,976 reads with an *N*_50_ value of 2,924 bp were obtained for the Nanopore library. After SPAdes assembly, contaminant contigs of <500 bp were removed using QUAST v5.0.2 (10). The assembled genome sequence is composed of 3 contigs, with an *N*_50_ value of 407,984 bp, a genome size of 739,863 bp, and a GC content of 26.7%. The genome is 98.7% complete according to the BUSCO analysis. Prokka annotated 1,349 protein-coding genes, 1 rRNA operon, and 27 tRNAs encoding every amino acid except tryptophan (although a tryptophan ligase gene was detected). This genome size is consistent with the 740- to 780-kbp value previously estimated using pulsed-field gel electrophoresis (1). *S. platyhelix* has an ANI of 77.3% compared to the fungus-growing ant metagenome-assembled genome (MAG) EntAcro10 (11). The EntAcro10 MAG is 100 kbp larger than that of *S. platyhelix*, and both strains possess genes for the transport and metabolism of arginine and glycerol, consistent with mutualistic insect-microbe relationships (11). However, *S. platyhelix* lacks the CRIPSR-cas1 locus found in the EntAcro10 MAG. The *S. platyhelix* genome sequence will facilitate future studies of *Mollicutes* evolution and the mechanisms of insect and plant symbioses.

### Data availability.

All data are available in NCBI under BioProject accession number PRJNA608455. The raw sequencing reads are deposited in the SRA under accession numbers SRX7849346 (2 × 250 bp), SRX7849347 (2 × 150 bp), and SRX7849348 (Nanopore). The assembled genome is deposited under accession number JAAVVK000000000.1.

## References

[1] Williamson DL, Adams JR, Whitcomb RF, Tully JG, Carle P, Konai M, Bové JM, Henegar RB 1997 *Spiroplasma platyhelix* sp. nov., a new mollicute with unusual morphology and genome size from the dragonfly *Pachydiplax longipennis*. Int J Syst Bacteriol 47:763–766. doi:10.1099/00207713-47-3-763. 9226909

[2] Sapountzis P, Nash DR, Schiøtt M, Boomsma JJ 2019 The evolution of abdominal microbiomes in fungus-growing ants. Mol Ecol 28:879–899. doi:10.1111/mec.14931. 30411820 PMC6446810

[3] Russell JA, Moreau CS, Goldman-Huertas B, Fujiwara M, Lohman DJ, Pierce NE 2009 Bacterial gut symbionts are tightly linked with the evolution of herbivory in ants. Proc Natl Acad Sci U S A 106:21236–21241. doi:10.1073/pnas.0907926106. 19948964 PMC2785723

[4] Tully JG, Rose DL, Whitcomb RF, Wenzel RP 1979 Enhanced isolation of *Mycoplasma pneumoniae* from throat washings with a newly modified culture medium. J Infect Dis 139:478–482. doi:10.1093/infdis/139.4.478. 374649

[5] Bolger AM, Lohse M, Usadel B 2014 Trimmomatic: a flexible trimmer for Illumina sequence data. Bioinformatics 30:2114–2120. doi:10.1093/bioinformatics/btu170. 24695404 PMC4103590

[6] Bankevich A, Nurk S, Antipov D, Gurevich AA, Dvorkin M, Kulikov AS, Lesin VM, Nikolenko SI, Pham S, Prjibelski AD, Pyshkin AV, Sirotkin AV, Vyahhi N, Tesler G, Alekseyev MA, Pevzner PA 2012 SPAdes: a new genome assembly algorithm and its applications to single-cell sequencing. J Comput Biol 19:455–477. doi:10.1089/cmb.2012.0021. 22506599 PMC3342519

[7] Seemann T 2014 Prokka: rapid prokaryotic genome annotation. Bioinformatics 30:2068–2069. doi:10.1093/bioinformatics/btu153. 24642063

[8] Seppey M, Manni M, Zdobnov EM 2019 BUSCO: assessing genome assembly and annotation completeness, p 227–245. *In* Kollmar M (ed), Gene prediction: methods and protocols. Springer, New York, NY. 10.1007/978-1-4939-9173-0_14 31020564

[9] Yoon S-H, Ha S-M, Lim J, Kwon S, Chun J 2017 A large-scale evaluation of algorithms to calculate average nucleotide identity. Antonie Van Leeuwenhoek 110:1281–1286. doi:10.1007/s10482-017-0844-4. 28204908

[10] Gurevich A, Saveliev V, Vyahhi N, Tesler G 2013 QUAST: quality assessment tool for genome assemblies. Bioinformatics 29:1072–1075. doi:10.1093/bioinformatics/btt086. 23422339 PMC3624806

[11] Sapountzis P, Zhukova M, Shik JZ, Schiøtt M, Boomsma JJ 2018 Reconstructing the functions of endosymbiotic Mollicutes in fungus-growing ants. Elife 7:e39209. doi:10.7554/eLife.39209. 30454555 PMC6245734

